# Dual expression of transgenic delta-5 and delta-6 desaturase in tilapia alters gut microbiota and enhances resistance to *Vibrio vulnificus* infection

**DOI:** 10.1371/journal.pone.0236601

**Published:** 2020-07-30

**Authors:** Keng-Yu Chiang, Wen-Chun Lin, Tsung-Yu Tsai, Cheng-Wei Lin, Shin-Jie Huang, Ching-Yu Huang, Sheng-Han Wu, Chuian-Fu Ken, Hong-Yi Gong, Jyh-Yih Chen, Jen-Leih Wu

**Affiliations:** 1 Department of Life Science, National Taiwan University, Taipei, Taiwan; 2 Institute of Cellular and Organismic Biology, Academia Sinica, Nankang, Taipei, Taiwan; 3 Marine Research Station, Institute of Cellular and Organismic Biology, Academia Sinica, Ilan, Taiwan; 4 Department of Aquaculture, National Taiwan Ocean University, Keelung, Taiwan; 5 Institute of Biotechnology, National Changhua University of Education, Changhua, Taiwan; 6 Center of Excellence for the Oceans, National Taiwan Ocean University, Keelung, Taiwan; National Cheng Kung University, TAIWAN

## Abstract

Omega-3 polyunsaturated fatty acids (n-3 PUFAs), such as eicosapentaenoic acid (EPA) and docosahexaenoic acid (DHA), exhibit antibacterial and anti-inflammatory activities. Furthermore, diets rich in n-3 PUFAs are known to improve disease resistance and limit pathogen infection in commercial aquaculture fishes. In this study, we examined the effects of transgenic overexpression of n-3 PUFA biosynthesis genes on the physiological response to bacterial infection in tilapia. We first established tilapia strains with single or dual expression of salmon delta-5 desaturase and/or delta-6 desaturase and then challenged the fish with *Vibrio vulnificus* infection. Interestingly, our data suggest that n-3 PUFA-mediated alterations in gut microbiota may be important in determining disease outcome via effects on immune response of the host. Both liver- and muscle-specific single and dual expression of delta-5 desaturase and delta-6 desaturase resulted in higher n-3 PUFA content in transgenic fish fed with a LO basal diet. The enrichment of n-3 PUFAs in dual-transgenic fish is likely responsible for their improved survival rate and comparatively reduced expression of inflammation- and immune-associated genes after *V*. *vulnificus* infection. Gut microbiome analysis further revealed that dual-transgenic tilapia had high gut microbiota diversity, with low levels of inflammation-associated microbiota (i.e., *Prevotellaceae*). Thus, our findings indicate that dual expression of transgenic delta-5 and delta-6 desaturase in tilapia enhances disease resistance, an effect that is associated with increased levels of n-3 PUFAs and altered gut microbiota composition.

## Introduction

The shared biosynthetic pathway of omega-3 polyunsaturated fatty acids (n-3 PUFAs) eicosapentaenoic acid (EPA, 20:5 n-3) and docosahexaenoic acid (DHA, 22: 6 n-3) utilizes α-linolenic acid (ALA, 18: 3 n-3) as a starting material and involves several desaturation and elongation steps that are performed by delta-4 desaturase, delta-5 desaturase, delta-6 desaturase, delta-5 elongase and delta-6 elongase [1]. In particular, delta-6 desaturase uses alpha-linolenic acid (ALA) as a substrate to produce stearidonic acid (SA), and delta-5 desaturase subsequently uses eicosatetraenoic acid (ETA) as a substrate to produce EPA. The EPA is then converted to DHA via elongation, desaturation, and beta-oxidation reactions [2]. The delta-6 desaturase enzyme has been shown to be rate limiting for the conversion of ALA to EPA in the lipid biosynthesis pathway [3]. Once synthesized, these and other n-3 PUFAs may function to improve animal health, at least partially as a result of their antibacterial and anti-inflammatory effects. In previous studies, bactericidal or bacteriostatic effects of DHA and EPA were demonstrated against *Propionibacterium acnes*, *Pseudomonas aeruginosa* and *Staphylococcus aureus* [4, 5]. Moreover, n-3 PUFAs are known to produce anti-inflammatory effects by reducing nuclear factor kappa B (NF-kB) activation [6]; NF-kB is a transcription factor that plays a critical role in activating inflammatory cytokines (e.g., IL-1, IL-2, IL-6, IL-12 and TNF-a), cyclooxygenase-2 (COX-2) and inducible nitric oxide synthase (iNOS) [7]. In particular, DHA and EPA were shown to inhibit lipopolysaccharide-induced activation of NF-kB in human kidney-2 cells [8], and feeding EPA to Atlantic salmon was found to reduce inflammatory response and protect against Atlantic salmon reovirus (ASRV) infection [9]. Thus, the ability to synthesize DHA and EPA may be highly beneficial to an organism’s defense against infection.

Recently, the influence of gut microbiota on a wide variety of physiological processes has become a topic of widespread interest. Gut microbes exist in a mutualistic relationship with the host, with the microbes exerting substantial control over critical physiological processes in the host, such as metabolism and immune response [10, 11]. The relationship between gut microbiota profiles and pathological conditions is under intense investigation, and beneficial microbiota are expected to be used to treat diseases in the near future [12]. Interestingly, accumulating evidence has suggested that n-3 PUFAs may be an important modulator of gut microbiota content. Some documented clinical effects of n-3 PUFA supplementation on gut microbiota composition include a decrease in the *Firmicutes/Bacteroidetes* ratio, decreased levels of *Coprococcus* and *Facecalibacterium*, and increased abundance of butyrate-producing bacteria [13, 14]. In a rodent study, it was found that fat-1 mice, which have high levels n-3 PUFAs, exhibit higher gut microbiota diversity and more abundant *Verrucomicrobia spp*. [15]. While many studies have demonstrated a relationship between n-3 PUFAs and gut microbiota, nearly all were performed in clinical populations or mouse models. Whether similar effects are also present in aquaculture fish has not been previously explored.

Tilapia is one of the most valuable aquaculture species and an important dietary constituent around the world. However, tilapia aquaculture practitioners often suffer economic losses due to pathogenic bacteria infections in the fish [16]. *Vibrio vulnificus* is one of the most detrimental bacterial pathogens in tilapia because it causes high mortality, mostly due to septicemia [17]. *V*. *vulnificus* stimulates inflammation in the host by activating Toll-like receptors (TLRs) and downstream signaling, i.e., NF-κB [18], which is diminished by n-3 PUFAs [6]. Since tilapia has low levels of EPA and DHA as compared to n-3 PUFA-rich salmon [19], elevation of n-3 PUFA levels in these fish would be expected to not only protect from bacterial infection but also to raise the nutritional value. In our previous studies, we successfully established liver- and muscle-specific expression of salmon delta-5 desaturase and delta-6 desaturase in zebrafish, which resulted in increased n-3 PUFA content and promoted antibacterial defenses [20, 21]. In this study, we applied the same expression system to aquaculture tilapia and challenged the transgenic fish with *V*. *vulnificus*. Dual expression of salmon delta-5 desaturase and delta-6 desaturase in tilapia increased the survival rate after *V*. *vulnificus* infection and diminished fish inflammation response. From a microbiome analysis, we also discovered that transgenic tilapia exhibit increased diversity of gut microbiota and decreased levels of inflammatory *Prevotellaceae* bacteria. Together, these findings suggest that increased n-3 PUFA levels in tilapia promote disease resistance and improve gut microbiota composition.

## Materials and methods

### Fish maintenance

Tilapia (*Oreochromis niloticus*) were obtained from a local aquaculture farm. Transgenic and wild-type fish were kept in the same fiberglass-reinforced plastic (FRP) tanks (120cm×120cm×70cm); different strains were separated by net cages (50cm×70cm×40cm) in recirculating aerated freshwater at 28°C, under a 12-h dark: 12-h light photoperiod. Each tank contained 20 tilapia, with an average body length of 10.0 ± 0.5 cm. Fish were hand fed with basal diet twice daily. Basal diet was obtained from United Aquaculture Feed (Kaohsiung, Taiwan). During the experimental period, all fish were fed with LO basal diet (at around 4% fish weight) and were measured weekly for 28 days. LO basal diet was made by supplementing basal diet with 3% linseed oil. Growth rate was monitored by body weight measurements. In all tilapia experiments, tricaine methanesulfonate (MS-222; Sigma) (150 mg/L and 600 mg/L) was used as an anesthetic and euthanasia agent. The animal protocols were approved by the Institutional Animal Care and Use Committee (IACUC) at Academia Sinica.

### Transgenic tilapia

Transgenic tilapia were established by utilizing two Tol2 transposon-mediated transgenesis vectors containing expression cassettes flanked by Tol2 transposase recognition elements [22]. The first vector contained Nile tilapia liver-specific *Fabp10a* (liver fatty acid binding protein 10 a) promoter or muscle-specific CKMb (creatine kinase, muscle b) promoter/enhancer [23] driving expression of a tetracycline-controlled transactivator (tTA). The second vector contained TcCFP13 (CFP) or enhanced eTcRFP11 (RFP) reporter genes isolated from Taiwan corals, which were bi-directionally expressed with delta-5 desaturase (Fadsd5) or delta-6 desaturase (Fadsd6) genes of Atlantic salmon (Salmo salar), respectively. The genes were under the control of a tetracycline responsive element (TRE) fused with CMV minimal (CMVmini) promoter, which is activated in liver or muscle by tTA transactivator expressed by the first vector. The synthesized Tol2 transposase messenger RNA (mRNA) were co-injected with three transgenesis vectors (liver- and muscle-specific tTA vectors and d5-desaturase/TcCFP13 or d6-desaturase/eTcRFP11) into one-cell stage tilapia embryos. The embryos were incubated in an egg tumbler incubator ZET-65 from ZissAqua at 28.5°C aquarium after microinjection. One transgenic tilapia founder was mated with one wild-type tilapia to generate the F1 generation; this mating scheme was used for both delta-5 desaturase and delta-6 desaturase transgenic tilapia. The dual expression of delta-5 desaturase and delta-6 desaturase in tilapia (dual-transgenic tilapia) was obtained by mating the F2 generation of delta-5 desaturase tilapia with the F2 generation of delta-6 desaturase transgenic fish. Wild-type and transgenic F2 progeny were used in all experiments. To check for germline transmission by the reporter gene, the transgenic tilapia were observed by fluorescence microscopy.

### Fatty acid extraction and analysis

To analyze the fatty acid compositions of fish food and tilapia, total lipids of fish food and tilapia liver tissue were extracted with organic solvent (chloroform: methanol, 2:1), using a method previously described by Folch *et al*. [24]. Lipids were saponified to produce potassium aliphatate by incubation at 100°C for 15 min in 2 mL 0.5 N methanol (Merck, Germany). After cooling the samples to room temperature, 2 mL 0.7 N HCl methanol and 1 mL BF3 (Boron trifluoride-methanol solution, 14% in methanol, Sigma) were added. The mixture was incubated at 100°C for 15 min to produce fatty acid methyl esters (FAME), followed by dilution with hexane (5 mg/mL). Samples were examined by Agilent 5975C Series GC-MSD (Agilent) under conditions described by Abu *et al*. [25].

### RNA isolation, RT-PCR and quantitative real-time RT-PCR

Liver and muscle tissues from five individual tilapia per group were thoroughly homogenized and used for RNA extraction, as described previously [21]. RNA concentration was quantified using a Nano-Drop spectrophotometer (Thermo, USA). Reverse transcription of RNA to cDNA was accomplished using the ReverTra Ace^®^ qPCR RT Master mix with gDNA Remover (TOYOBO, Japan), according to the manufacturer’s recommendations. The RNA samples were denatured at 65°C for 5 min, and immediately cooled on ice. Then, 2 μL RNA template (200 ng/μL) was added to 2 μL of 4X DN Master Mix and 4 μL nuclease-free water. The mixture was incubated at 37°C for 5 min. The solution was mixed with 5X RT Master Mix II, and reverse transcription was performed as follows: sample was placed at 37°C for 15 min, 50°C for 5 min, and 98°C for 5 min. For each reverse transcription-polymerase chain reaction (RT-PCR), reagents included 2.5 μL 10x PCR buffer (Invitrogen), 2 μL dNTP (2.5 mM) (Invitrogen), 1 μL MgCl2 (50 mM) (Invitrogen), 1 μL primer mix (forward primer and reverse primer, 10 μM), 0.1 μL Platinum Taq Polymerase (5 U/μL) (Invitrogen), 2 μL cDNA and 16.4 μl ddH2O. The PCR reaction condition was set as follows: pre-denaturation, 94°C for 1 min; denaturation, 94°C for 30 sec; annealing, 55°C for 30 sec; elongation, 72°C for 40 sec; and A-tailing 72°C for 10 min. The three steps of denaturation, annealing, and elongation were repeated for 35 cycles. The following primers were used: RT-D5-For, 5’- GTGTTTCGTAAAGATCCTGATATCAACTCA-3’; RT-D6-For, 5’- GTGTTGAGTAAAGATCCTGATGTTAATATG-3’; RT-D56-Rev, 5’- AGAGGAGCCACCAGGTGGTAGTTATGAC-3’. The PCR products were analyzed by agarose gel electrophoresis. The resultant cDNA was used as a template for quantitative real time polymerase chain reaction (real-time PCR) by an Applied Biosystems StepOne Plus Real-Time PCR system (ABI, USA) and SYBR^®^ Green Realtime PCR Master Mix (TOYOBO, Japan), following the manufacturer’s protocol. The reaction mixture was composed of 2.2 μL ddH2O, 5 μL SYBR^®^ Green Realtime PCR Master Mix, 2 μL cDNA, and 0.4 μL (10 mM) of each specific primer pair (Table 1). PCR was performed under the following conditions: 95°C for 20 sec; 40 cycles of 95°C for 3 sec and 60°C for 30 sec; 95°C for 15 sec to detect a melting curve; 60°C for 1 min; and 95°C for 15 sec. The final results were calculated as relative expression of each gene normalized to the house-keeping gene, *EF1α*.

**Table 1:** Primers used in this study.

Primer name	Sequence(5’→3’)	Accession
***Ef-1α* (F)**	TCAACATCGTGGTCATTGG	AB075952
***Ef-1α* (R)**	CTCAGCCTTCAGTTTGTCC	
***Il-1β* (F)**	TCAGTTCACCAGCAGGGATG	DQ061114
***Il-1β* (R)**	GACAGATAGAGGTTTGTGCC	
***Il-6* (F)**	AGATGTCCACTGTCAAGCC	XM005478656
***Il-6* (R)**	ACCGAGTAGATGAGCAGACC	
***Il-8* (F)**	TGCCACACTGAAAAGGAC	NM001279704
***Il-8* (R)**	AGTCATCTCGTGAAAGGAAC	
***Il-10* (F)**	GCTGCTAGATCAGTCCGTCG	KP645180
***Il-10* (R)**	GGACTCCACGTGAGGCTTTA	
***Mcp-8* (F)**	CGGGTTAGCTGTTGGCATTGT	XM005478749
***Mcp-8* (R)**	AAGCAAGCAGAGAAAACCACTTCA	
***β-defensin* (F)**	TCGTGTGGTTGTTTTGGC	KF294753
***β-defensin* (R)**	AGCCCAGAGGTCCAAAGAAC	

### Western blot

Total protein of liver and muscle tissue was extracted in 500 μL 2-D rehydration sample buffer, according to the protocol for the Ready-Prep Protein Extraction kit (Bio-Rad). Concentrations of protein were determined using a protein assay kit (Bio-Rad). For western blot, proteins (100 μg/well) were separated on a 4–12% NuPAGE Bis-Tris Mini Gel (Invitrogen). Proteins were transferred to a PVDF membrane, which was then blocked with 5% BSA for 1 h. Membranes were rinsed with PBST buffer and then incubated with primary anti-V5 antibody (1:1000; Sigma) overnight at 4°C; GAPDH antibody (1:10000; Millipore) was used as a positive control. Membranes were washed with PBST three times for 10 min and incubated with secondary antibody for 1 h at room temperature, followed by repeated PBST wash steps. Signals were detected using Immobile Western Chemiluminescent HRP Substrate (Millipore) and captured with an Imaging System (UVP).

### *Vibrio vulnificus* culture

*V*. *vulnificus* (strain 93U204) was cultured on BHI (Brain heart infusion powder, BD DifcoTM) agar plates containing 1.5% w/v sodium chloride (Merck) at 30°C for 16 h. A single colony of *V*. *vulnificus* was subsequently selected to incubate in 300 mL BHI (NaCl 1.5%) media at 28°C on a shaker for a further 16 h. Subsequently, 30 μL bacteria were cultured with 300 μL of BHI (NaCl 1.5% w/v) media at 28°C on a shaker to obtain the appropriate concentration (~10^7^ CFU/mL).

### *Vibrio vulnificu*s challenge

The experimental wild-type and F2 transgenic tilapia were about 3 months of age with an average 10 cm body length. A total of four tanks (20cm×30cm×20cm), each holding 10 L of aerated water, were used to maintain the tilapia; the fifteen tilapia from each group were used in the bacterial challenge study. The tilapia were starved for one day and then anesthetized by MS-222 (150 mg/L) before intraperitoneal injection (IP-injection). The tilapia were infected by IP-injection of 30 μL *V*. *vulnificus* (1.8 × 10^6^ CFU/ mL, diluted with PBS). The survival rates of 15 tilapia from each group of wild-type, delta-5-, delta-6-, and dual-transgenic tilapia were calculated after *V*. *vulnificus* infection for 72 h. Three independent trials were performed. After IP-injection, tilapia behavior was closely monitored, and all the moribund tilapia were euthanized with MS-222 (600 mg/L); moribund behaviors included, lost activity, imbalance, surfacing, and ascites. Tilapia mortality was recorded at 0, 3, 6, 12, 24, 48, and 72 h after challenge. To count colony-forming units, the liver tissues from five individual tilapia per group infected with *V*. *vulnificus* were homogenized in BHI (NaCl 1.5%, 1 mg/20 μL) media, and the supernatants were serially diluted in BHI (NaCl 1.5%, 1 mg/20 μL) media. For each dilution, 50 μL was spotted onto a TCBS (Thio- sulfate-citrate-bile salts-sucrose, powder, BD DifcoTM) plate. The TCBS Plates were incubated at 28°C for 16 h, and colonies were counted to calculate the colony-forming units (CFU)/mL. Three independent trials were performed.

### DNA extraction and sequencing of gut microbiota

The hindguts of wild-type and transgenic tilapia were collected for gut microbiota sequencing. Bacteria total genomic DNA from hindgut was extracted using QIAamp PowerFecal DNA Kit (QIAGEN, Germany), as described previously by Hart *et al*. [26]. The variable regions of 16S rRNA were used to distinguish between bacterial species. Specifically, the V3-V4 conserved regions of intestinal bacteria were examined. The sequencing of gut microbiota in tilapia was performed and analyzed by BIOTOOLS company (Taiwan), following procedures described previously by Chen *et al*. [27]. The 16S rRNA V3–V4 regions were amplified using a specific primer set (Forward Primer: CCTAYGGGRBGCASCAG, Reverse Primer: GGACTACNNGGGTATCTAAT). The PCR amplicons (466 bp) were sequenced on an Illumina HiSeq 2500 platform, and the sequences were analyzed to identify the operational taxonomic units (OTUs). Paired-end reads were analyzed and merged using FastQC and FLASH (V1.2.7). Quality filtering on the raw tags was performed according to split_libraries_fastq.py. The tags were detected and chimera sequences were removed using UCHIME. OTU clusterings were picked based on >97% sequence similarity using UPARSE software (Version 7.0.1001). OTU annotations were made according to the Greengenes Database (Version 2.2). The final analysis outputs, including alpha diversity, beta diversity and PLS-DA (Partial Least Squares Discriminant Analysis), were used to compare bacterial diversity between samples.

### Statistical analysis

The data are presented as average ± standard deviation (SD) or average ± standard error of the mean (SEM), as indicated. Statistical analysis was performed by one-way analysis of variance (ANOVA) followed by Welch’s test. Significance was set at *P* < 0.05.

## Results

### Tissue-specific expression of desaturases in tilapia

**Fig 1:**
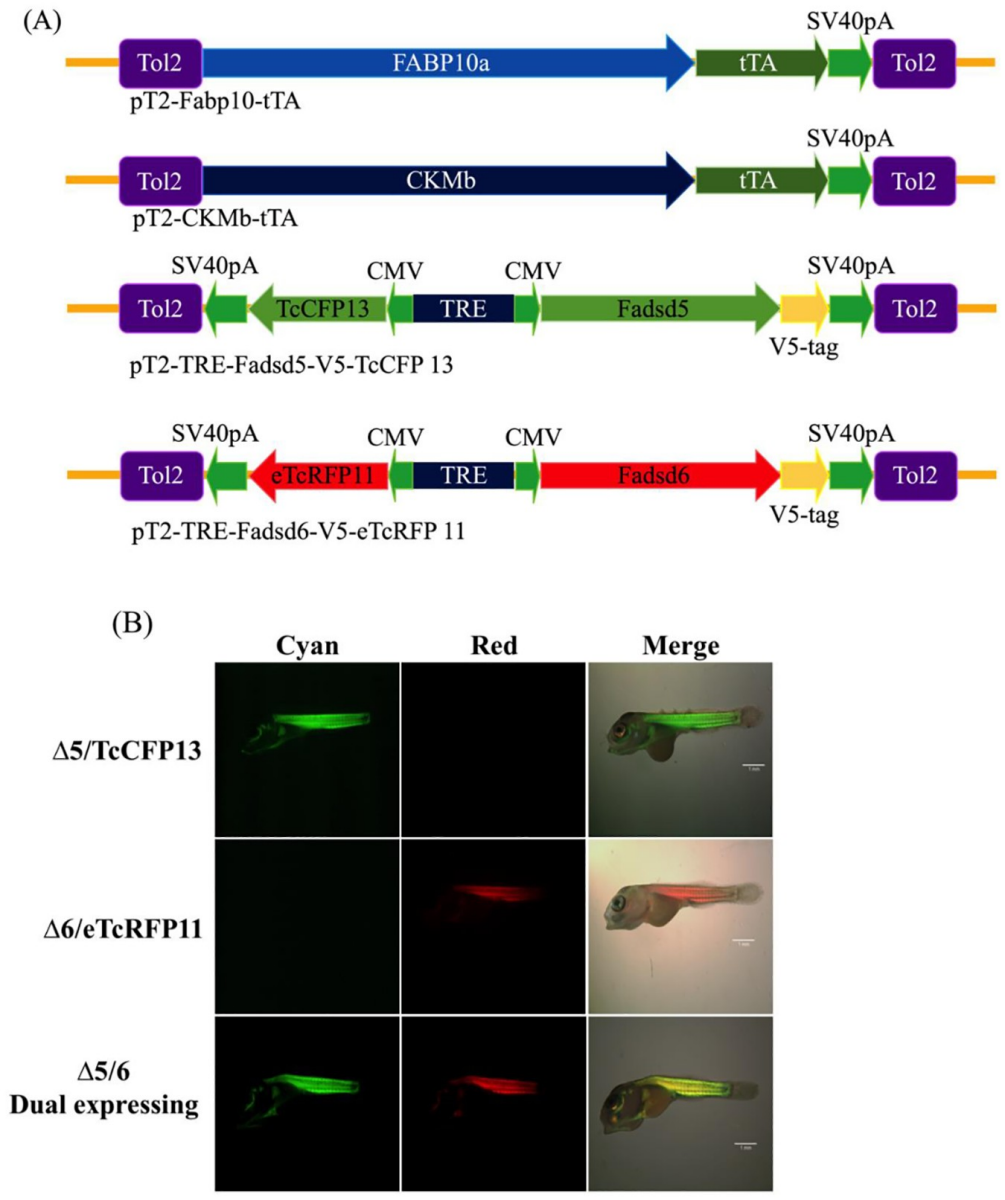
Establishment of tissue-specific *Fadsd5*- and *Fadsd6*-transgenic tilapia (A) Schematics show the liver- and muscle-specific tetracycline transactivator (tTA) plasmid (pT2-Fabp10-tTA and pT2-CKMb-tTA) and two tetracycline-responsive expression plasmids (pT2-TRE-fadsd5-V5-TcCFP13 and pT2-TRE-fadsd6-V5-eTcRFP11). All expression plasmids include the Tol2 transposon. (B) Fluorescence microscopy images of delta-5 (cyan), delta-6 (red), and delta-5/delta-6 dual-transgenic tilapia (both cyan and red). Scale bar, 1mm.

To increase the content of n-3 PUFAs in tilapia, salmon biosynthesis enzymes, delta-5 desaturase, and delta-6 desaturase, were overexpressed as transgenes. Transgenic tilapia were established by utilizing two Tol2 transposon-mediated transgenesis vectors. The first vector contains Nile tilapia liver-specific *fabp10a* promoter or muscle-specific *CKMb* promoter/enhancer that drive expression of a tTA. The second vector contains TcCFP13 or enhanced eTcRFP11 reporter genes that are bi-directionally expressed with delta-5 desaturase or delta-6 desaturase genes of Atlantic salmon, respectively; the genes are under the control of a TRE fused with CMV promoter, which is activated in liver or muscle by tTA transactivator expressed by the first vector (Fig 1A). The synthesized Tol2 transposase mRNAs were co-injected with three transgenic vectors (including liver- and muscle-specific tTA vectors and d5-desaturase/TcCFP13 or d6-desaturase/eTcRFP11) into one-cell stage tilapia embryos, leading to specific expression of delta-5 and delta-6 desaturases in liver and muscle tissues.

**Fig 2:**
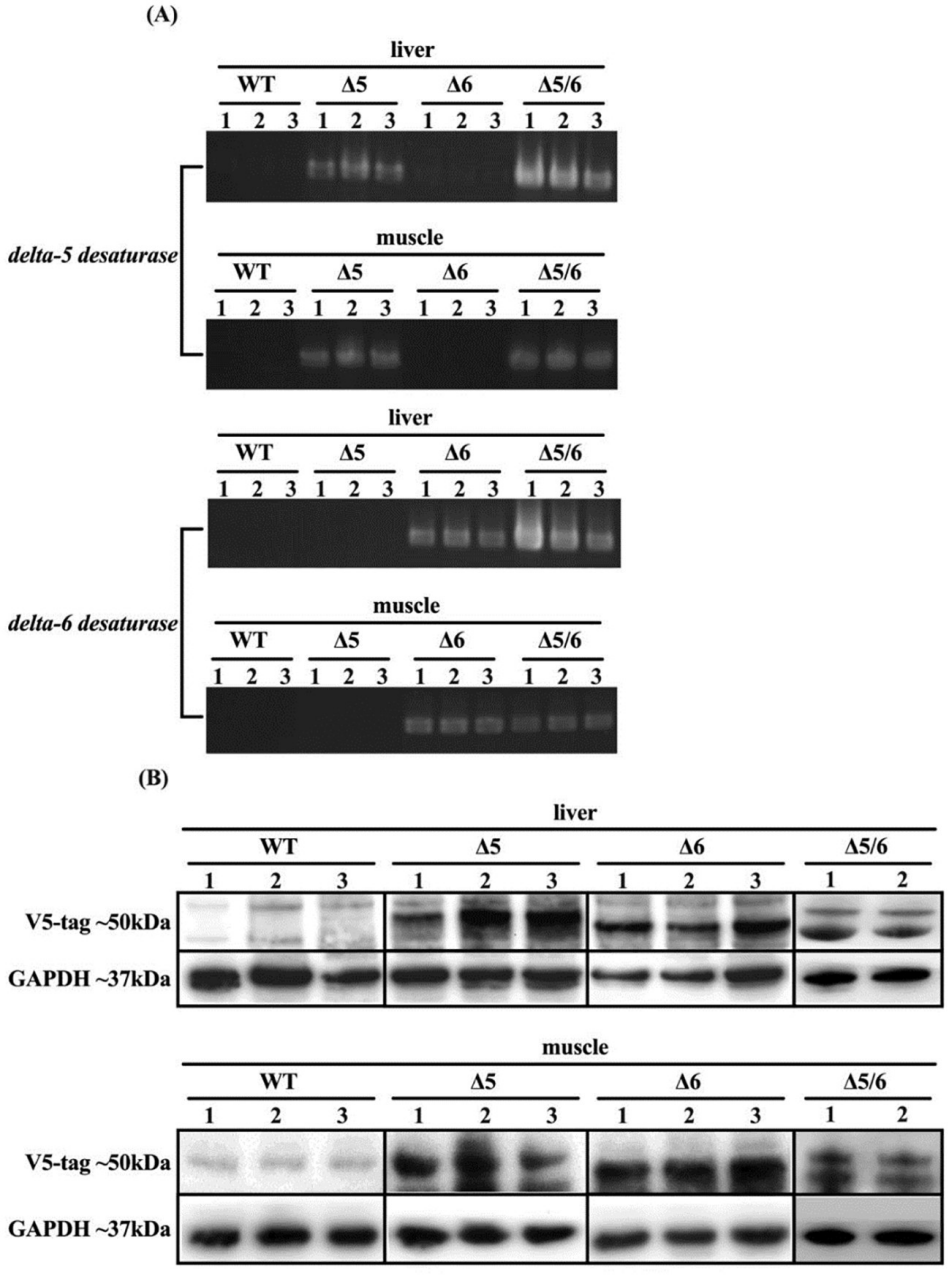
Exogenous delta-5 and delta-6 desaturase expression in liver and muscle from transgenic tilapia. (A) The exogenous gene expression of *Fadsd5* (delta-5) and *Fadsd6* (delta-6) were detected by RT-PCR from liver and muscle tissue in wild-type and transgenic tilapia. Western blots were performed to detect V5-tagged delta-5 and delta-6 desaturase in wild-type and transgenic tilapia. Mouse GAPDH was used as an internal control.

Cyan and red fluorescence reporter signals were observed in muscle tissue of the delta-5 desaturase and delta-6 desaturase transgenic lines, respectively. The delta-5 desaturase transgenic line showed only cyan fluorescence, and the delta-6 desaturase transgenic line showed only red fluorescence; the delta-5 desaturase and delta-6 desaturase dual-transgenic line showed both cyan and red fluorescence (Fig 1B). Exogenous delta-5 and delta-6 gene expression was further detected in transgenic fish liver and muscle. Primer sets, RT-D5-For/RT-D56-Rev and RT-D6-For/RT-D56-Rev, are targeted to the exogenous delta-5 and delta-6 desaturase gene, respectively, and both amplify a PCR product of 529 bp (base pairs). In each tilapia strain, three individuals were examined. The RT-PCR results showed that exogenous delta-5 desaturase and delta-6 desaturase could be respectively detected in delta-5 and delta-6 desaturase single-transgenic tilapia. The dual-transgenic tilapia exhibited both delta-5 and delta-6 desaturase gene (Fig 2A). The expression levels of V5-tagged delta-5 and delta-6 desaturase proteins (~50 kDa) were measured in liver and muscle tissue of transgenic fish by western blotting. V5-tagged delta-5 and delta-6 protein were observed in liver and muscle tissue of delta-5 and delta-6 transgenic tilapia. The V5-tagged delta-5 protein (upper band) and V5-tagged delta-6 protein (lower band) were both expressed in dual-transgenic fish (Fig 2B). GAPDH (~37 kDa) was used as an internal control. Thus, our results show that the fluorescence reporters are reliable indicators of exogenous delta-5 and delta-6 desaturase expression in both liver and muscle.

### Fatty acid compositions of basal diet and supplemented linseed oil

**Fig 3:**
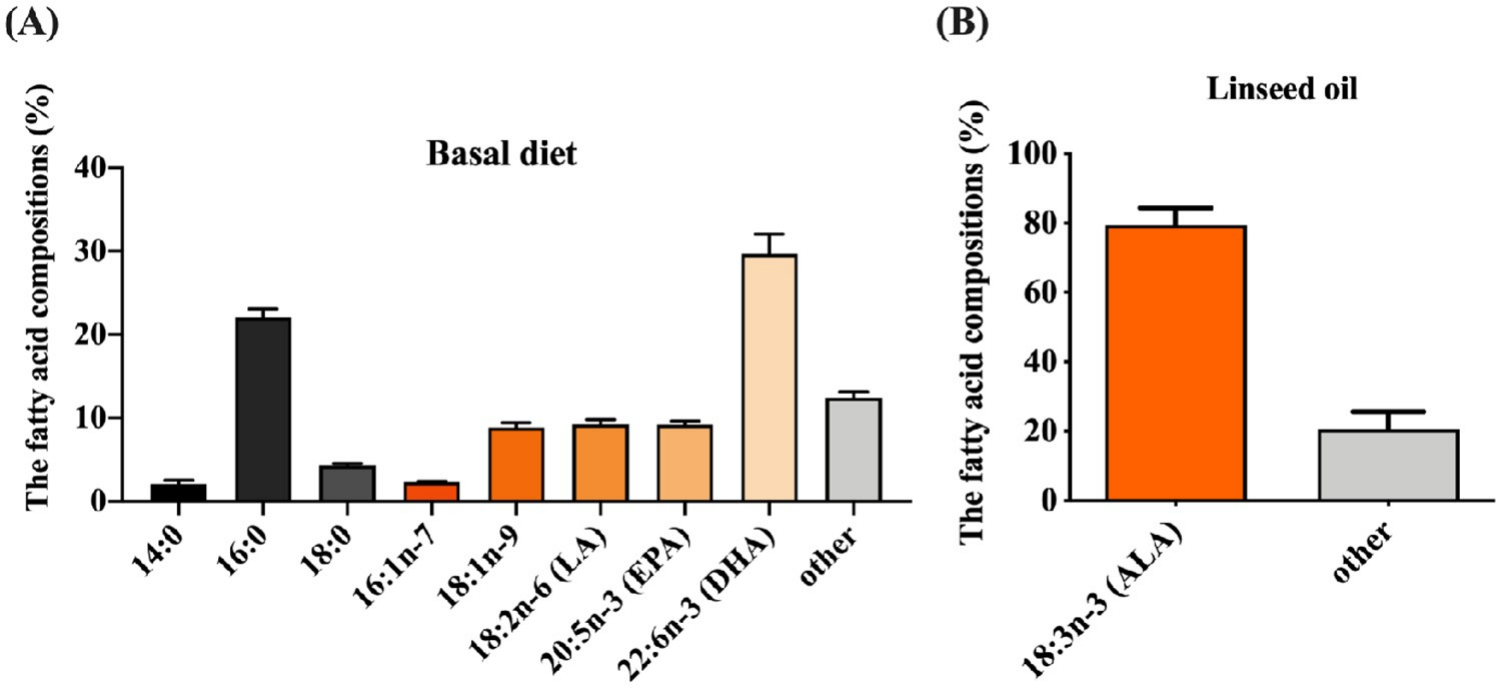
Fatty acid composition of basal diet and linseed oil. (A) Fatty acid composition of basal diet. (B) Percentage of ALA (18:3 n-3) of supplemented linseed oil. Measurements were made by gas chromatography with mass spectrometry (GC-MS).

To increase biosynthesis of n-3 PUFAs in the transgenic tilapia, the fish were fed with LO basal diet that was composed of basal diet and 3% linseed oil. The fatty acid composition of the basal diet was analyzed by gas chromatography with mass spectrometry (GC-MS). The analysis showed that 29.7% of the fatty acid was DHA (22:6 n-3), 9.2% was EPA (20:5 n-3), 9.2% was palmitic acid (LA, 18:2 n-6), 8.9% was oleic acid (18:1 n-9), 2.3% was palmitoleic acid (16:1 n-7), 4.3% was 18:0, 22.1% was 16:0, 2% was 14:0 and 12.4% was other fatty acids (Fig 3A). Approximately 79.4% of the linseed oil was ALA (18:3 n-3), the starting material for synthesis of DHA and EPA (Fig 3B). Thus, the LO basal diet was rich in n-3 and n-6 fatty acids and could provide sufficient substrate for PUFA biosynthesis.

### LO basal diet increased the DHA content in dual-transgenic tilapia

**Fig 4:**
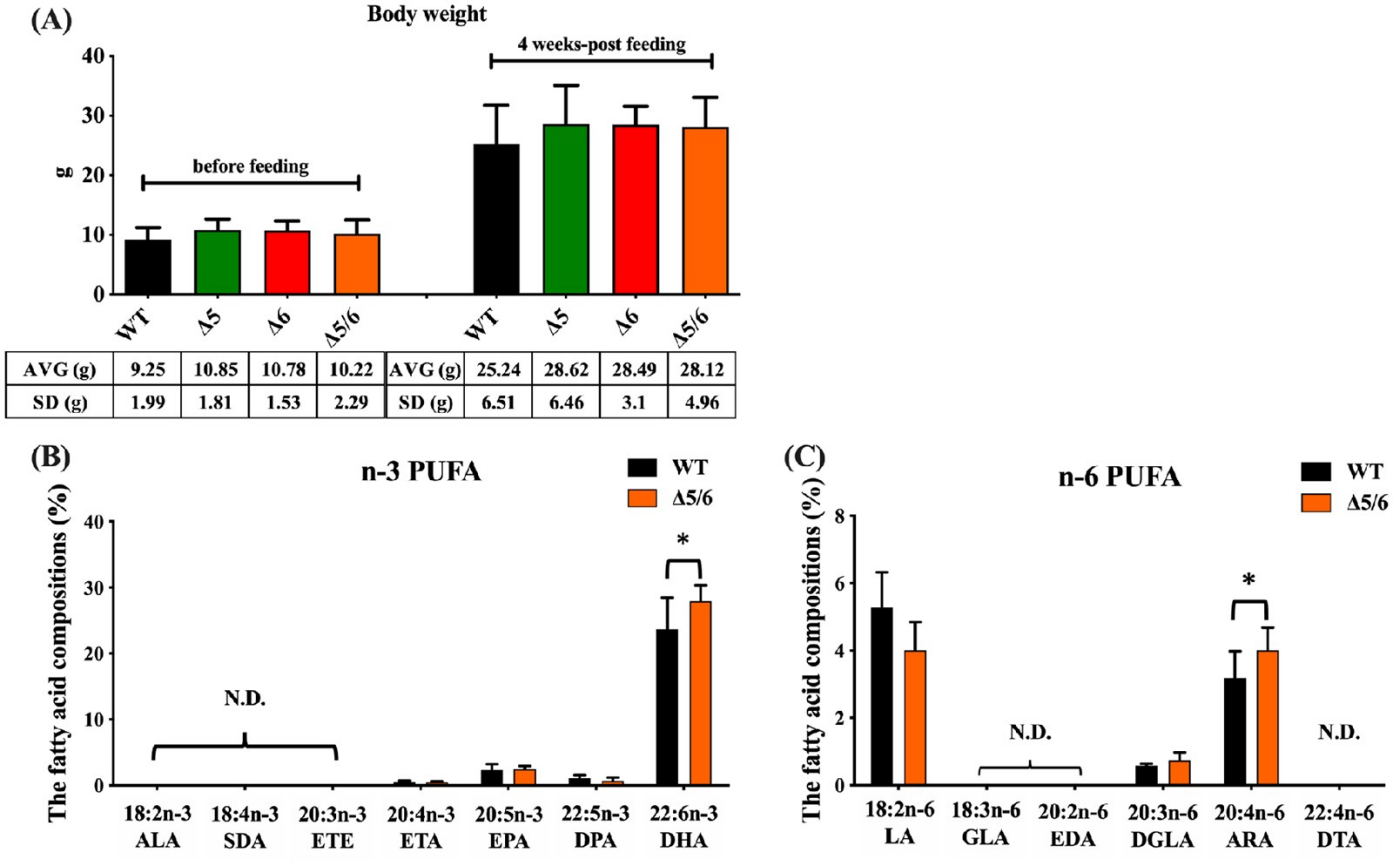
Body weight, n-3, and n-6 polyunsaturated fatty acid content analysis in wild-type and transgenic tilapia. (A) Body weights of wild-type, delta-5, delta-6, and dual-transgenic tilapia before and after feeding on fish food for 28 days are shown as the mean ± SD (n = 20). g, gram; AVG, average; SD, standard deviation. (B) n-3 polyunsaturated fatty acid content in wild-type and dual-transgenic tilapia, as examined by GC-MS (n = 8). ALA, α-linolenic acid; SDA, stearidonic acid; ETE, eicosatrienoic acid; ETA, eicosatetraenoic acid; EPA, eicosapentaenoic; DPA, docosapentaenoic acid; DHA, docosahexaenoic acid. (C) n-6 polyunsaturated fatty acid content in wild-type and dual-transgenic tilapia, as examined by GC-MS (n = 8). LA, linoleic; GLA, γ-linolenic acid; EDA, eicosadienoic acid; DGLA, Dihomo-γ-linolenic acid; ARA, arachidonic acid; DTA, adrenic acid. N.D., not detected. Significance was set at *P* < 0.05, as determined by Welch’s t-test.

Tilapia body weights were measured before and after feeding with LO basal diet for around 4% fish weight per day for 28 days. There was no major difference in average weights of wild-type (25.24 ± 6.51 g) and dual-transgenic tilapia (28.12 ± 4.96 g) after feeding with the LO basal diet for 28 days. The weights of transgenic tilapia expressing only liver-specific delta-5 desaturase (28.62 ± 6.46 g) or delta-6 desaturase (28.49 ± 3.1 g) were also not obviously different than wild-type (Fig 4A). Therefore, we concluded that the expression of exogenous desaturase does not alter body weight.

We next examined the lipid composition of the transgenic tilapia liver after feeding with LO basal diet. The total lipid content was not different between wild-type and dual-transgenic tilapia (data not shown). As expected, DHA content was 1.2-fold higher in delta-5 and delta-6 desaturase dual-transgenic tilapia than in wild-type tilapia; the difference was significant according to Welch’s t-test (Fig 4B). However, we did not find significant differences in other n-3 PUFAs, including EPA, eicosatetraenoic acid (ETA) and docosapentaenoic acid (DPA), between wild-type and dual-transgenic tilapia. On the other hand, the n-6 PUFA, arachidonic acid (ARA) was significantly elevated by 1.3-fold in dual-transgenic fish compared to wild-type tilapia (Fig 4C). Other n-6 PUFAs, including linoleic acid (LA) and dihomo-γ-linolenic acid (DGLA) did not show significant differences between wild-type and dual-transgenic tilapia. In summary, we found evidence that n-3 and n-6 PUFAs were upregulated in dual-transgenic tilapia fed on a LO basal diet.

### Dual-transgenic tilapia had improved survival after *V*. *vulnificu*s challenge

**Fig 5:**
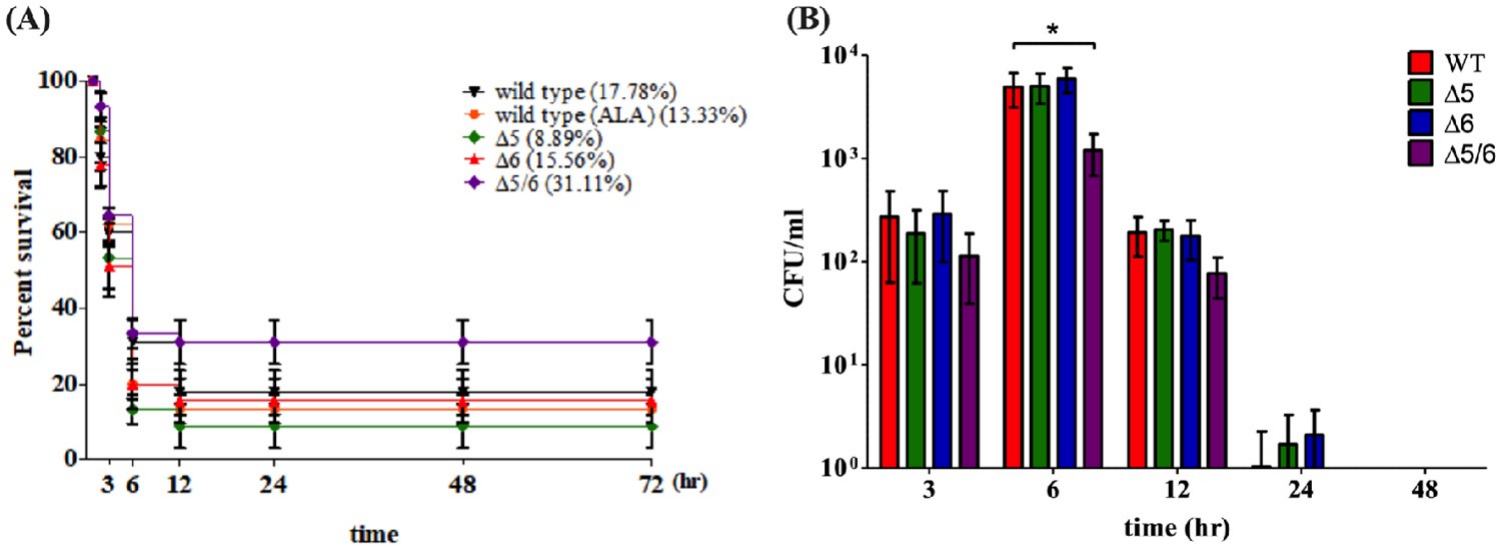
Survival rates and fatty acid compositions of wild-type and transgenic tilapia after challenge with *V*. *vulnificus* for 72 h. (A) Survival rates of wild-type and three transgenic lines IP-injected with *V*. *vulnificus*. Wild-type (black), delta-5 (green), delta-6 (red), and dual-transgenic tilapia (purple) were fed on LO basal diet before *V*. *vulnificus* challenge. Wild-type-ALA (orange) was fed on only ALA linseed oil before *V*. *vulnificus* challenge. (B) Bacterial contents were examined in tilapia after *V*. *vulnificus* infection. Liver Tissues were collected and homogenates were cultured on TCBS agar plates. The bacterial counts (CFU/mL) were assessed for wild-type, delta-5, delta-6, and dual-transgenic tilapia. Values are presented as mean ± SEM. Significance was determined by t-test (*P < 0.05).

Since the dual-transgenic tilapia exhibited elevated n-3 and n-6 PUFA levels, we next examined whether the fish had enhanced resistance to *V*. *vulnificus* infection. Wild-type and transgenic tilapia received intraperitoneal injections of 30 μL *V*. *vulnificus* (1.8 × 10^6^ CFU/mL), and fish mortality was assessed at 0, 3, 6, 12, 24, 48, and 72 h after injection. The survival rates of all groups of tilapia were between 50 and 65% at 3 h after *V*. *vulnificus* challenge. The survival rate of wild-type tilapia was 17.78%, and for wild-type tilapia fed with ALA diet, survival was 13.33% at 72 h after infection. On the other hand, the dual-transgenic fish exhibited the highest survival rate of all groups at 31.11% at 72 h post-injection (Fig 5A). Surprisingly, the delta-5 and delta-6 desaturase single-transgenic tilapia had survival rates lower than wild-type, with 8.89% and 15.56%, respectively surviving at 72 h. We then examined whether *V*. *vulnificus* growth was affected by desaturase expression in transgenic tilapia. *V*. *vulnificus* was collected at 3, 6, 12, 24, and 48 h after injection from tilapia tissue homogenates and re-cultured on a TCBS-agar plate. As expected, the *V*. *vulnificus* CFU counts from dual-transgenic tilapia were significantly lower than those from wild-type at 6 h after injection (Fig 5B). Moreover, the CFU counts from dual-transgenic tilapia were slightly decreased at 3 and 12 h after injection. These results suggest that high PUFA content in dual-transgenic tilapia could protect the fish against *V*. *vulnificus* by suppressing growth of the pathogen.

### Inflammatory gene expression after *V*. *vulnificus* infection was attenuated in transgenic tilapia

**Fig 6:**
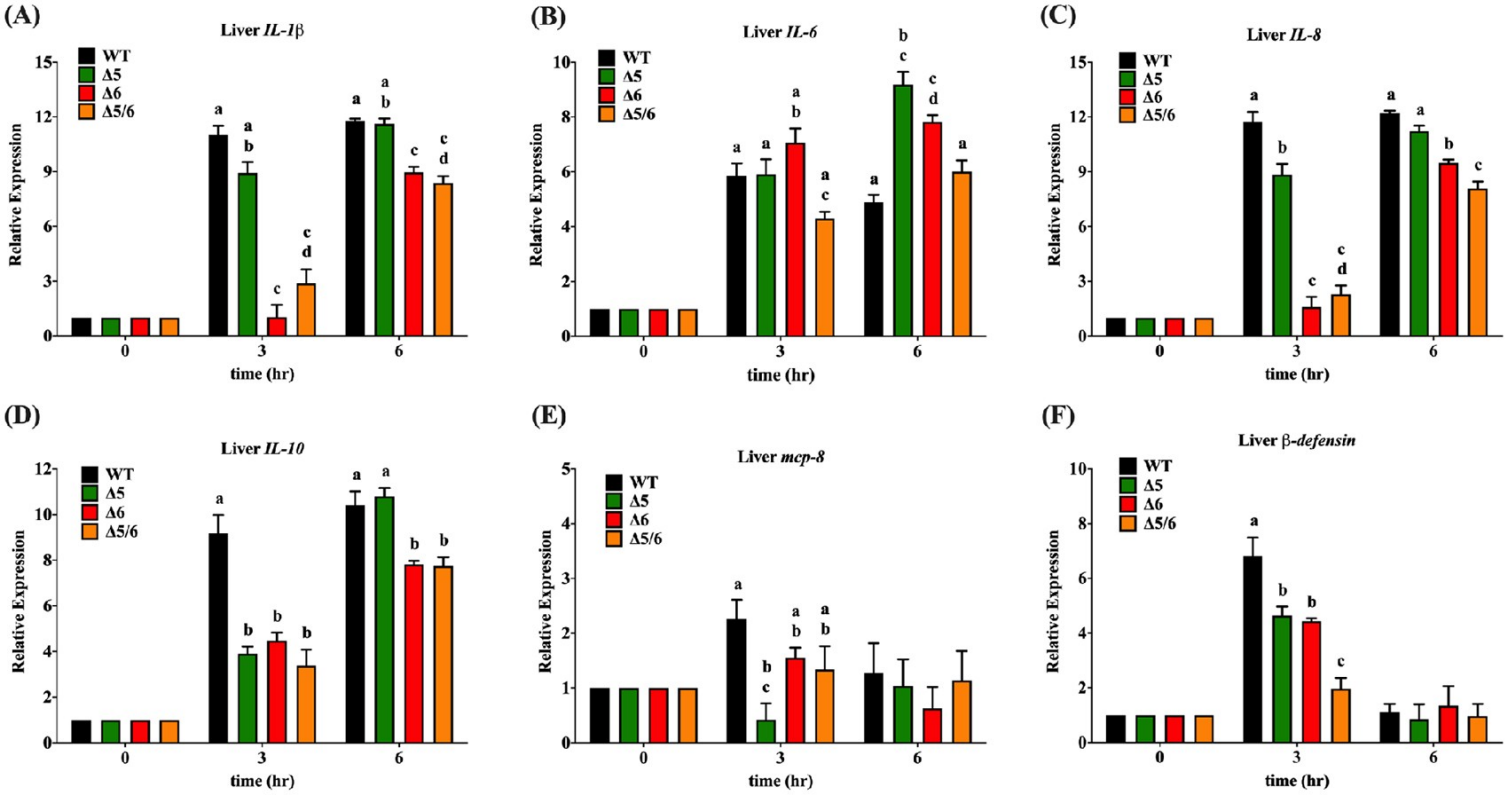
Inflammatory and immune-related gene expression in liver tissue of wild-type and transgenic tilapia at the indicated times after challenging with *V*. *vulnificus* challenge. Gene expression was examined by quantitative PCR. (A) *IL-1β*, (B) *IL-6*, (C) *IL-8*, (D) *IL-10*, (E) *Mcp-8*, (F) *β-defensin*. Expression level are presented as mean ± SEM. Significance was set at *P* < 0.05, as determined by one-way ANOVA followed by Duncan’s test. Assignment of different letters indicates a significant difference between expression levels.

**Fig 7:**
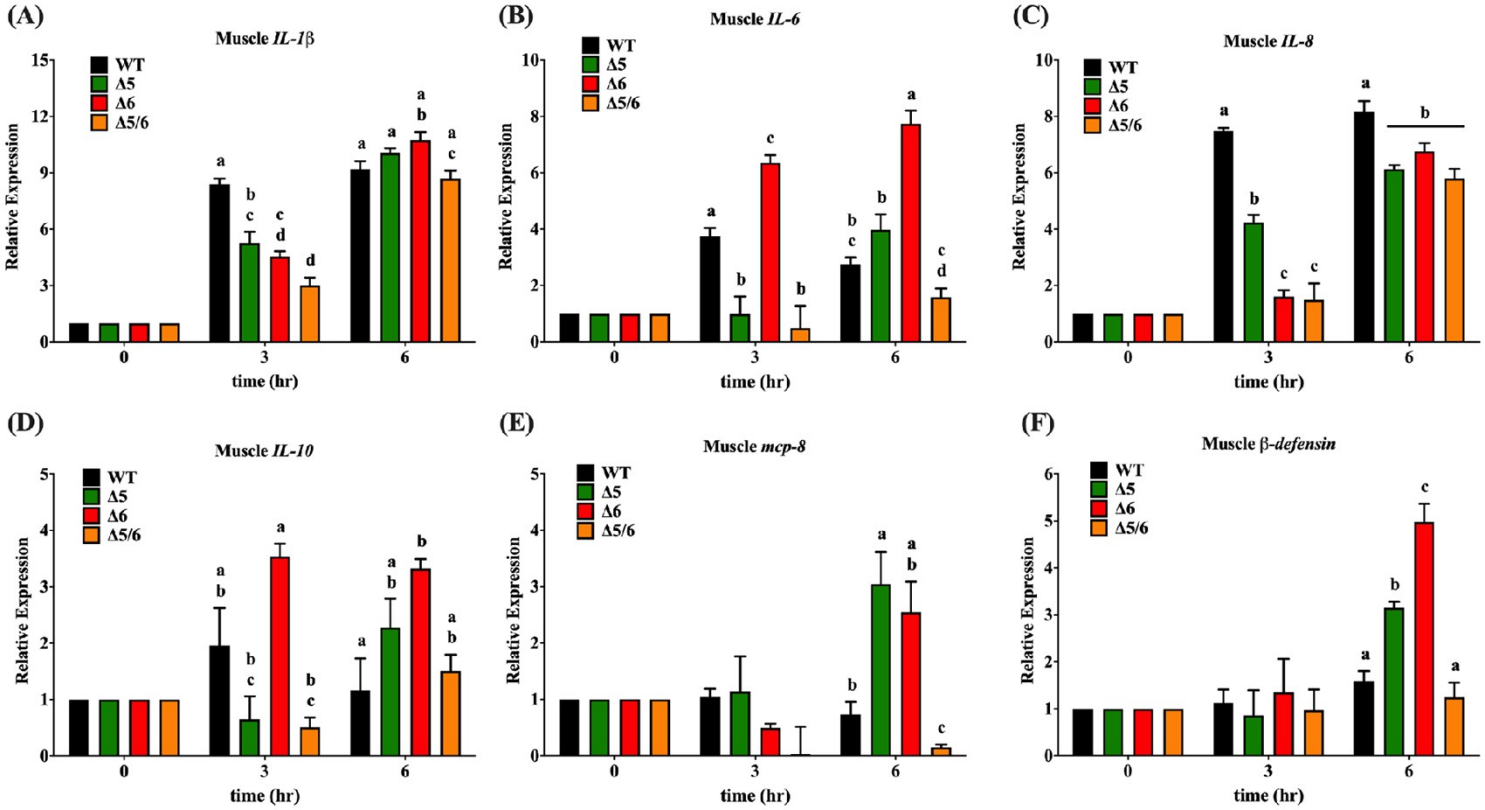
Inflammatory and immunity related gene expression in muscle tissue of wild-type and transgenic tilapia at the indicated times after challenging with *V*. *vulnificus*. The gene expression was examined by real-time PCR. (A) *IL-1β*, (B) *IL-6*, (C) *IL-8*, (D) *IL-10*, (E) *Mcp-8*, (F) *β-defensin*. Expression levels are presented as mean ± SEM. Significance was set at *P* < 0.05, as determined by one-way ANOVA followed by Duncan’s test. Assignment of different letters indicates a significant difference between expression levels.

To elucidate the gene expression response of transgenic tilapia after *Vibrio vulnificus* infection, inflammation- and immune response-related gene products were quantified from liver and muscle by real-time PCR after bacterial challenge. The inflammation response began with dramatic activation of the interleukin family gene in wild-type fish. Liver *IL-1β* expression in dual-transgenic tilapia was 3.8-fold lower and 1.4-fold lower than in wild-type tilapia at 3 h and 6 h post-infection, respectively (Fig 6A); muscle *IL-1β* expression in dual-transgenic tilapia was 2.8-fold lower than in wild-type tilapia at 3 h after injection (Fig 7A). The muscle IL-6 expression in dual-transgenic tilapia was 7.7-fold lower than in wild-type tilapia at 3 h after injection (Fig 7B). However, the expression level of *IL-6* in dual-transgenic tilapia liver showed no significant difference than in wild-type tilapia (Fig 6B). In the liver, significantly lower expression of inflammation-related genes, including *IL-8 and IL-10*, also could be observed in dual-transgenic tilapia at 3 h and 6 h post-infection. The expression of liver *IL-8* in dual-transgenic fish was 5.1-fold lower and 1.5-fold lower than in wild-type fish at 3 h and 6 h after injection, respectively; the expression of liver *IL-10* in dual-transgenic fish was 2.7-fold lower and 1.3-fold lower than in wild-type fish at 3 h and 6 h after injection, respectively (Fig 6C and 6D). Muscle *IL-8* expression in dual-transgenic tilapia was 5.0-fold lower and 1.4-fold lower than in wild-type tilapia at 3 h and 6 h post-infection, respectively (Fig 7C). Nevertheless, there was no significant difference in muscle *IL-10* expression between wild-type and dual-transgenic tilapia after *V*. *vulnificus* infection (Fig 7D).

On the other hand, expression of *Mcp-8*, which is known to play a critical role in resistance to *Streptococcus agalactiae* infection in tilapia [28], was not noticeably different between wild-type and dual-transgenic tilapia liver (Fig 6E), and a 4.9-fold lower detection level was observed in dual-transgenic tilapia muscle at 6 h post-infection (Fig 7E). Furthermore, expression of *β-defensin*, an antibacterial peptide that participates in both tilapia and human immunity response [29, 30], was also examined. As excepted, expression of liver *β-defensin* in dual-transgenic tilapia was 3.5-fold weaker than wild-type tilapia at 3 h post-infection (Fig 6F). However, the expression of muscle *β-defensin* in dual-transgenic tilapia showed no significant difference than in wild-type tilapia after infection (Fig 7F). Based on these results we conclude that the low expression levels of pro-inflammatory and immunity-associated genes in dual-transgenic tilapia liver and muscle tissue may indicate PUFA-mediated suppression of the inflammation response after *Vibrio vulnificus* infection.

### High bacterial diversity and decrease of inflammation-associated gut microbiota in transgenic tilapia

**Fig 8:**
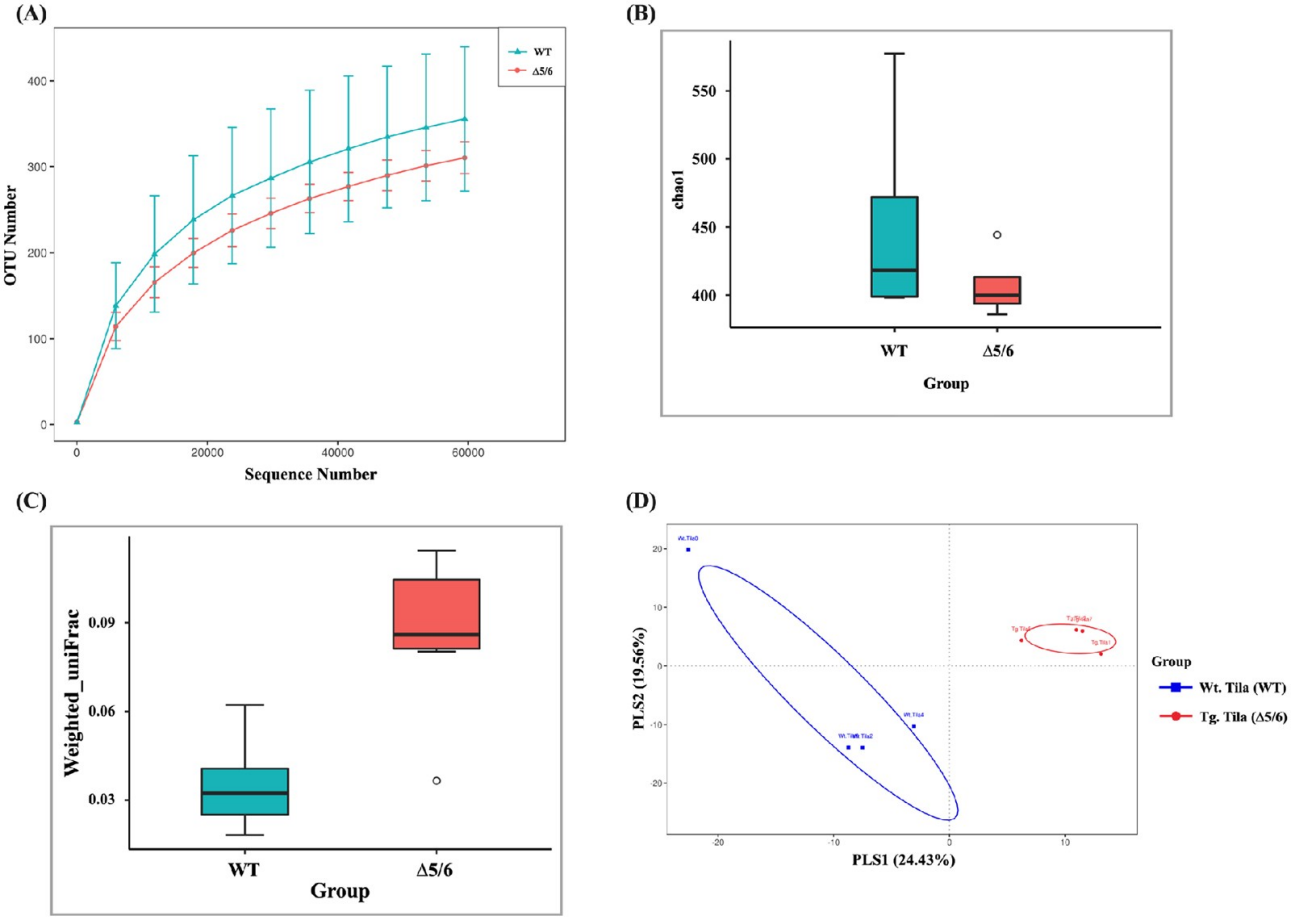
Bacterial diversity in dual-transgenic tilapia. (A) The rarefaction curve in alpha diversity, (B) chao1 richness in alpha diversity, (C) weighted uniFrac in beta diversity, and (D) PLS-DA plots collected from wild-type and dual-transgenic tilapia. n = 4 per group.

We also investigated whether the elevation of n-3 PUFAs might affect the composition of gut microbiota in transgenic tilapia after LO basal diet feeding because gut microbiota composition is associated with n-3 PUFAs and might be involved in inflammation. The alpha-diversity rarefaction curve indicated that sufficient sampling depth was achieved in both wild-type and transgenic tilapia (Fig 8A). The chao1 index further showed that bacterial richness was not different between wild-type and transgenic tilapia (Fig 8B). However, the bacterial beta-diversity showed an ‘a’ value increase in the transgenic fish compared with wild type (Fig 8C), meaning that the bacterial diversity of transgenic fish is higher than that of wild type. A partial least squares discriminant analysis (PLS-DA) was performed to calculate the variation of gut microbiota. The PLS-DA plot shows that PLS 1 is 24.43% and PLS 2 is 19.56% of variation of gut microbiota composition. A significant separation in PLS 1 and PLS2 values between wild-type and transgenic tilapia was observed (Fig 8D).

**Fig 9:**
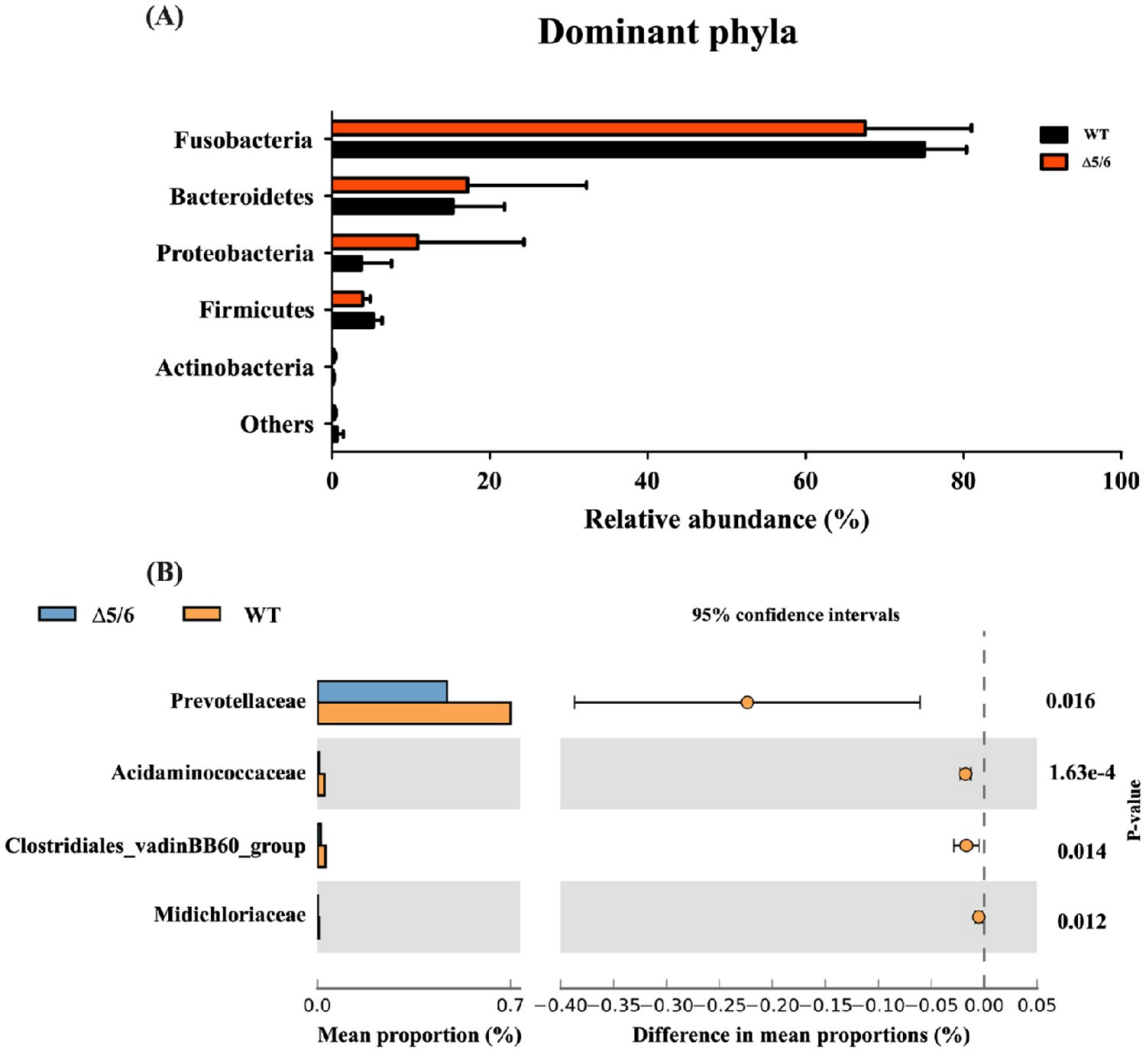
Abundance of gut microbiota in dual-transgenic tilapia. Gut microbiota contents were compared between wild-type and dual-transgenic tilapia at the (A) phylum and (B) family levels. n = 4 per group.

Next, we analyzed the abundance of certain bacterial taxa to clarify the role of n-3 PUFAs in gut microbiota. The results of the phylum analysis demonstrated no significant differences between wild-type and transgenic tilapia. *Fusobacteria*, *Bacteroidetes*, *Proteobacteria and Firmicutes* were the dominant strains in all lines of tilapia (Fig 9A). However, we did observe that transgenic tilapia had significantly reduced abundance of the *Prevotellaceae* (Family) (*P* < 0.05) (Fig 9B). *Prevotellaceae* is anaerobic Gram‐negative bacteria that exists in the healthy human body, but increased *Prevotella* abundance is positively associated with inflammatory disorders [31]. Thus, the transgenic tilapia showed high bacterial diversity and significantly decreased levels of *Prevotellaceae*.

## Discussion

Aquiculture practices can benefit from advances that increase the nutritional value of products and reduce economic losses to fisheries. According to Food and Agriculture Organization of the United Nations (FAO) statistics, tilapia is one of most important fish in aquaculture, and worldwide production has steadily increased every year. However, tilapia fisheries often suffer from infections with bacteria, such as *S*. *agalactiae* [32] and *V*. *vulnificus*. Previous studies have suggested the potential of n-3 PUFAs to increase resistance of aquiculture fish to bacterial infection [4, 5]. In addition, n-3 PUFAs exhibit anti-bacterial effects against *V*. *alginolyticus* [21] and *V*. *vulnificus* [20] in a zebrafish model. Therefore, we expected that elevation of n-3 PUFA content in tilapia could increase both antimicrobial resistance and nutritional value in tilapia. Our strategy was to transgenically express exogenous desaturases in tilapia and feed the fish with a LO basal diet that is supplemented with linseed oil.

We generated transgenic tilapia that express *Fadsd5* and *Fadsd6* in the liver and muscle under respective control of the *Fabp10a* and *CKMb* promoters. The reason we chose these tissues is because the liver and muscle are the major organs for lipid metabolism. In particular, liver functions in lipid biosynthesis, and muscle is important for lipid storage [33]. Hence, we expected that liver and muscle specific-transgenic fish will enhance PUFA biosynthesis and accumulation. The fatty acid analysis in dual-transgenic tilapia indicated that DHA content was 1.2-fold higher than that in wild-type tilapia. This elevation of n-3 PUFAs was in agreement with previous studies in zebrafish [20, 21]. Moreover, in our study, we also observed an increase in n-6 PUFA content, probably because n-3 and n-6 PUFAs are coordinately biosynthesized and compete for delta-6 desaturase substrate [34]. Both n-3 and n-6 PUFAs are important for maintaining physiological homeostasis, but a recent study showed that excessive n-6 PUFAs may lead to health disorders and promote inflammatory response [35–37]. Notably, a previous report revealed that the *fat-1* gene plays a critical role in converting n-6 PUFAs to n-3 PUFAs [38], and transgenic *fat-1* mice were successfully established for the purpose of enriching n-3 PUFA content [39]. Consequently, we plan to establish transgenic *fat-1* tilapia to convert n-6 PUFAs to n-3 PUFAs in future work.

The DHA content in dual delta-5 and delta-6 desaturase-expressing transgenic fish was higher than wild-type fish. After *V*. *vulnificus* infection, the survival rate of dual-transgenic fish was increased, and *V*. *vulnificus* growth was diminished in dual-transgenic fish. Together, these results suggest that the high DHA content in transgenic fish may have antibacterial effects on *V*. *vulnificus*. Similar benefits of DHA have been reported in many studies, as DHA is known to increase expression of antimicrobial peptides [40]. Moreover, in our analysis of inflammation-associated genes, *IL-1β*, *IL-8* and *IL-10* were significantly lower at early stages of *V*. *vulnificus* infection both in liver and muscle tissue of dual-transgenic fish compared to wild type. It was previously reported that DHA at high concentrations can decrease pro-inflammatory cytokine production, especially IL-1β, at initial priming step of inflammation [41, 42]. Therefore, the transgenic delta-5 and delta-6 desaturase tilapia resistance to *V*. *vulnificus* infection and inhibition of proinflammatory response may be due to the increased levels of DHA.

Many studies have demonstrated the gut microbiota content in tilapia is affected by diet, stress and environmental factors (water, salinity, pH, etc.) [43–45]. The gut microbiota composition in n-3 PUFA-rich transgenic tilapia was evaluated in this study. From our microbiome analysis, we found the bacterial diversity of transgenic fish is higher than that in wild type. This result is similar to previous studies that showed feeding an n-3-PUFA-rich diet increased bacterial diversity in human participants [46]. In tilapia and other freshwater fishes, *Fusobacteria* and *Proteobacteria* generally account for the major proportion of gut microbiota [47], but our microbiome analysis revealed *Fusobacteria* and *Bacteroidetes* were the top two dominant strains in both wild-type and transgenic tilapia. We suppose the proportions of bacteria are dependent on the rearing conditions. In further taxonomic analysis, the relative abundances of gut bacteria at the phylum-level were not different between wild-type and transgenic tilapia. However, further family-level analysis showed the level of *Prevotellaceae* was significantly lower in transgenic tilapia. The *Prevotellaceae* are anaerobic Gram‐negative bacteria of the *Bacteroidetes* phylum which also exist in humans. Many studies have found that increased *Prevotellaceae* abundance is related to the chronic inflammatory disease, such as irritable bowel syndrome [48]. One study indicated *Prevotella* induces immune response in periodontitis by regulating IL-1α and IL-1β levels [49]. *In vitro* studies further suggested that *Prevotella* display an enhanced capacity to induce inflammatory factors, such as IL-6, IL-8 and tumor necrosis factor-α (TNF-α) [50]. From our Q-PCR data, we found the level of inflammatory genes, *IL-1β*, *IL-6* and *IL-8*, are lower in transgenic fish than wild types after *V*. *vulnificus* infection. We speculate the inhibition of inflammatory response in n-3 PUFA-rich transgenic fish might be mediated by the decreased proportion of inflammation-associated *Prevotellaceae* bacteria. According to the previous study, DHA has been shown to prevent inflammation and bacterial dysbiosis by changing the cytokines production, macrophages recruitment, especially the composition of the intestinal microbiota and the intestine integrity [51]. This study suggests that DHA is highly correlated with the composition of intestinal microbiota, especially those associated with inflammation. However, more studies to clarify the relationships between n-3 PUFA-regulated inflammation and gut microbiota will be essential.

## Supporting information

S1 Raw images (PDF)
