# Supplementary material for: Dual expression of transgenic delta-5 and delta-6 desaturase in tilapia alters gut microbiota and enhances resistance to Vibrio vulnificus infection
Source: PLoS One. 2020 Jul 30;15(7):e0236601. doi: 10.1371/journal.pone.0236601 (PMC7392239; doi:10.1371/journal.pone.0236601)
Supplement: S1 Raw images — (PDF) [file pone.0236601.s001.pdf]

Exogenous salmon delta-5 desaturase gene expression was performed by RT-PCR from liver and muscle tissue in wild-type, delta-5 desaturase, delta-6 desaturase, and dual-transgenic tilapia independently. In each tilapia strain, three individuals were detected by RT-PCR.

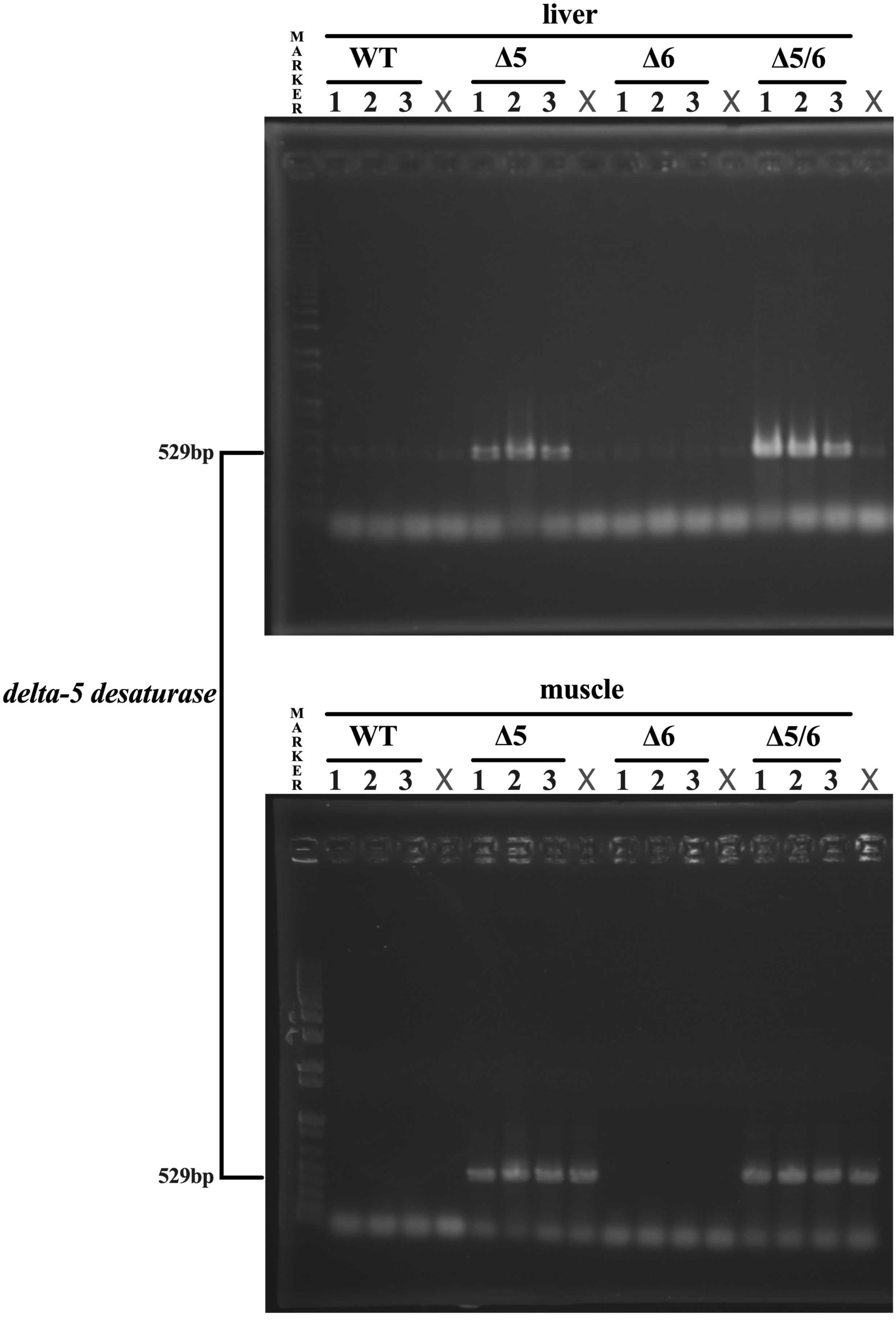

detal-5 desaturase RT-PCR of Figure 2A was genetated from the original images

Exogenous salmon delta-6 desaturase gene expression was performed by RT-PCR from liver and muscle tissue in wild-type, delta-5 desaturase, delta-6 desaturase, and dual-transgenic tilapia independently. In each tilapia strain, three individuals were detected by RT-PCR.

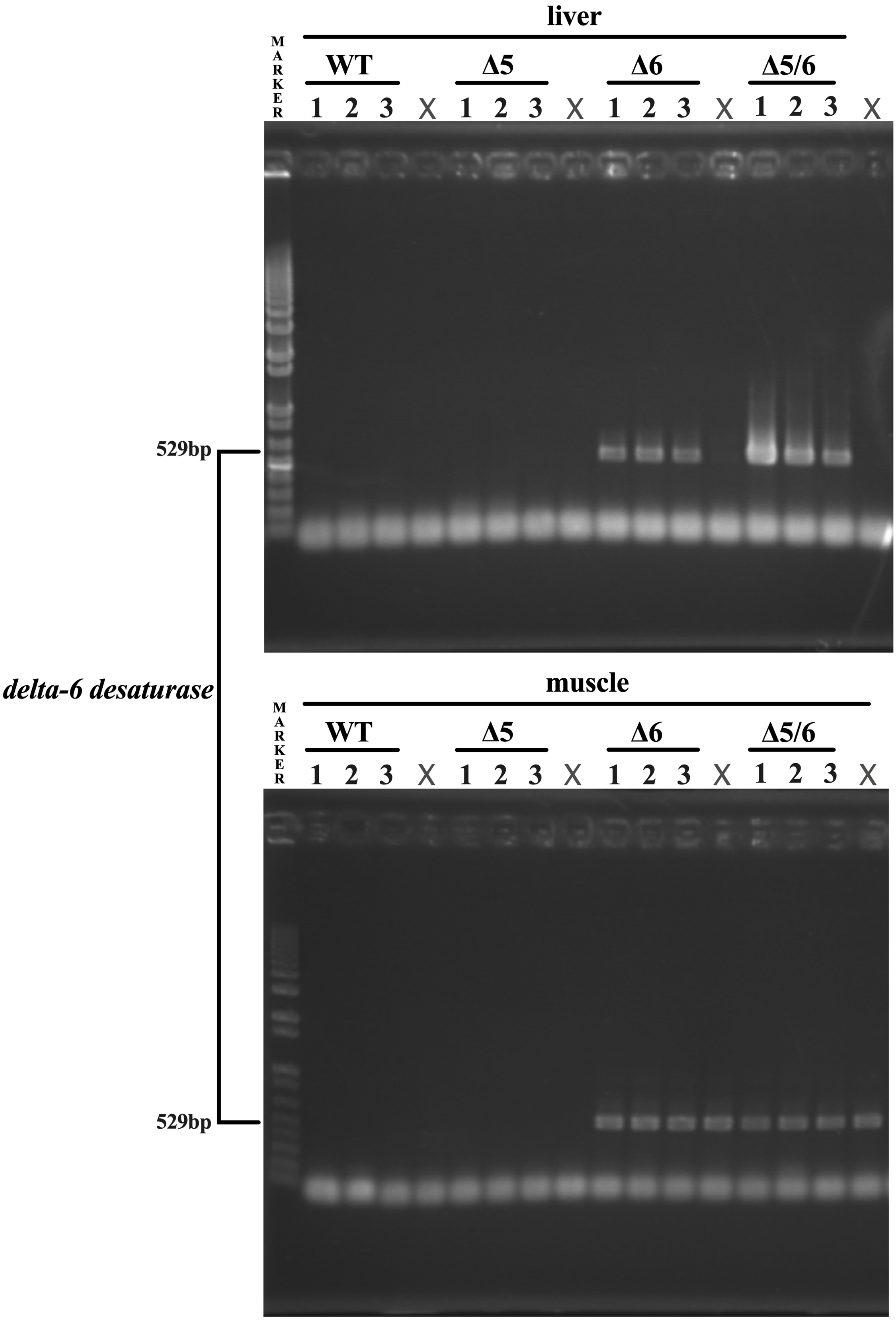

detal-6 desaturase RT-PCR of Figure 2A was genetated from the original images

Western blots were performed to detect V5-tagged delta-5 and delta-6 desaturase from liver tissue in wild-type, delta-5 desaturase, delta-6 desaturase, and dual-transgenic tilapia independently. Mouse GAPDH was used as an internal control. Three individuals of wild-type, delta-5, and delta-6 transgenic tilapia and two dual transgenic tilapia were detected respectively by Western blots.

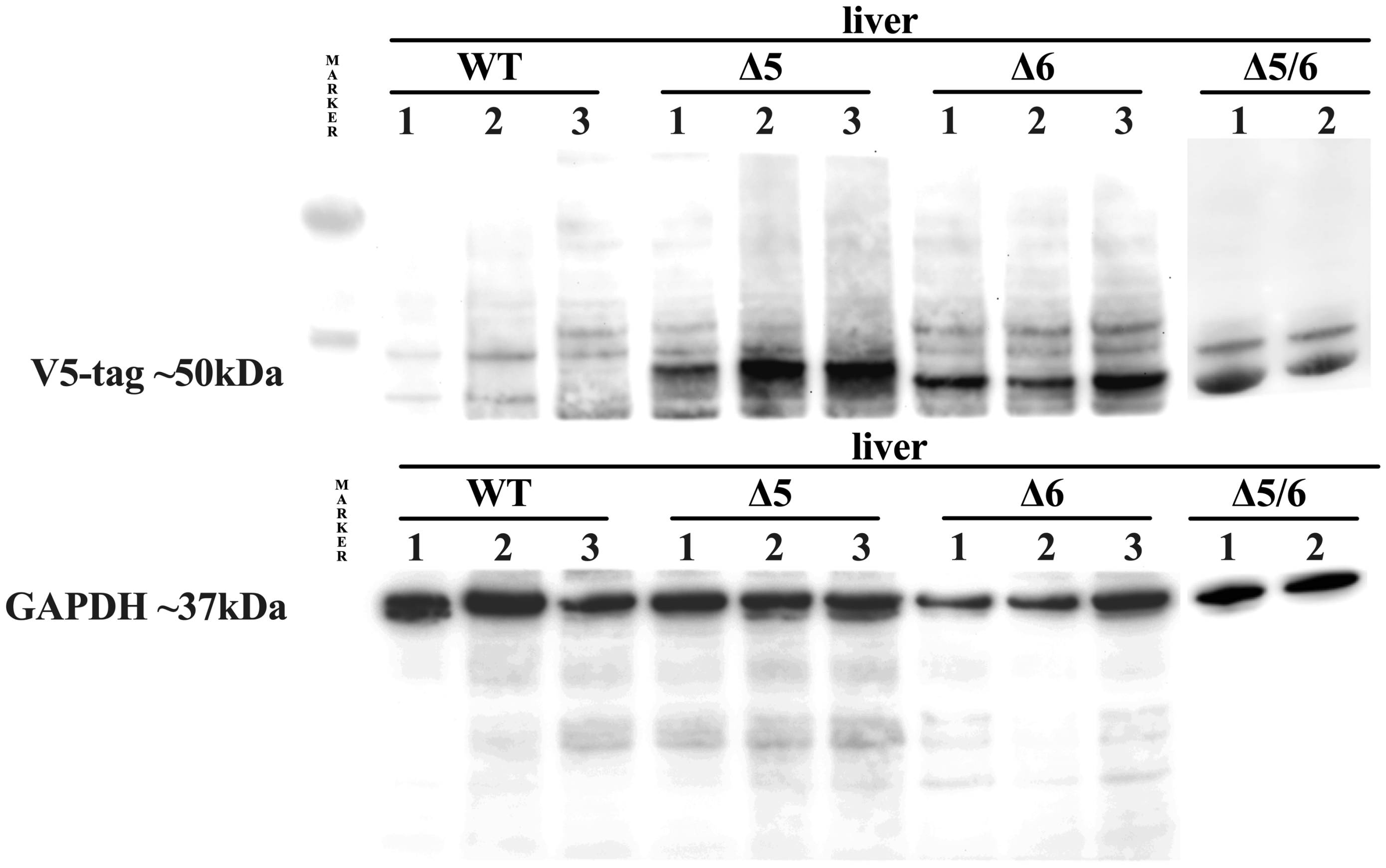

Liver part of Figure 2B was genetated from the original images

Western blots were performed to detect V5-tagged delta-5 and delta-6 desaturase from muscle tissue in wild-type, delta-5 desaturase, delta-6 desaturase, and dual-transgenic tilapia independently. Mouse GAPDH was used as an internal control. Three individuals of wild-type, delta-5, and delta-6 transgenic tilapia and two dual transgenic tilapia were detected respectively by Western blots.

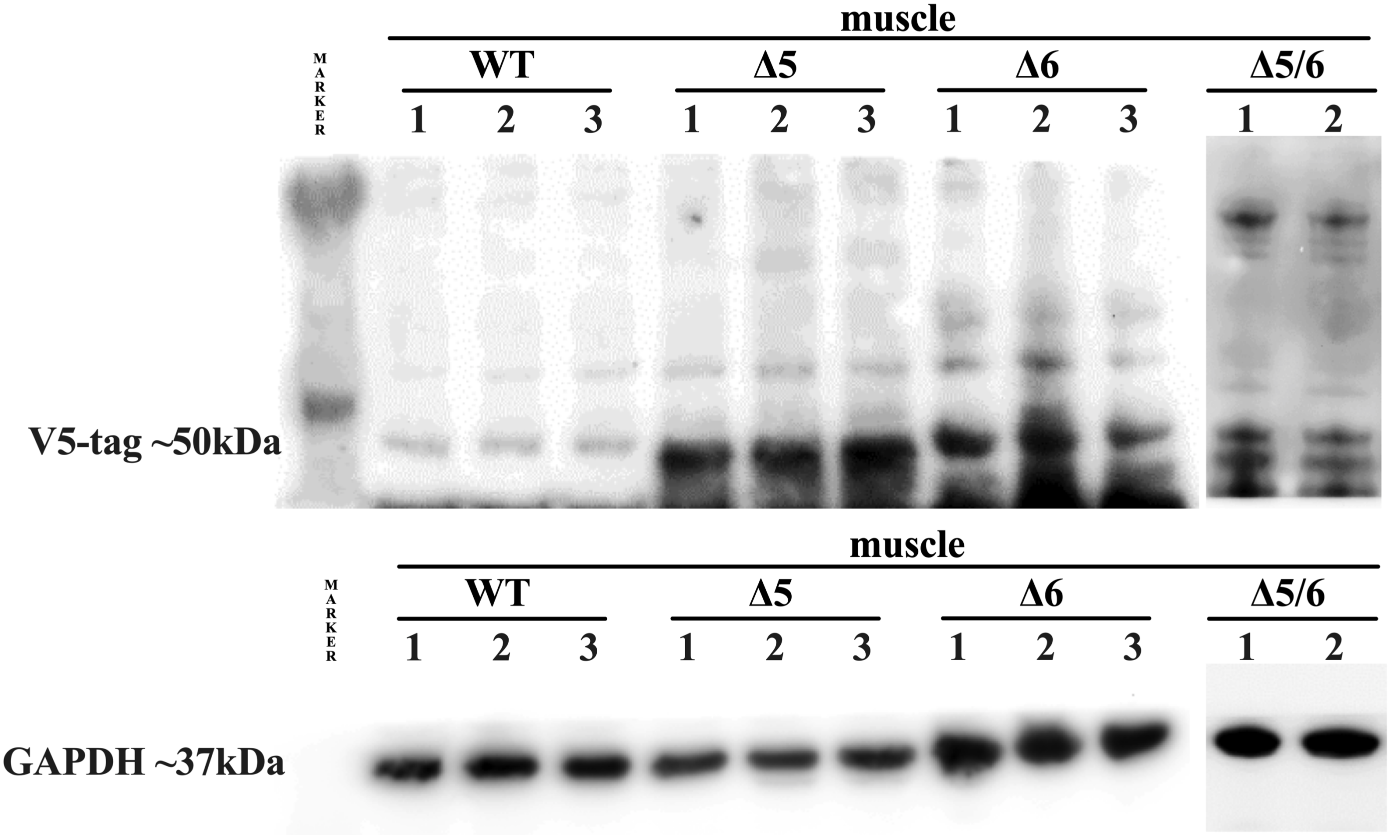

Muscle part of Figure 2B was genetated from the original images
